# The Effects of 1 Egg per Day on Iron and Anemia Status among Young Malawian Children: A Secondary Analysis of a Randomized Controlled Trial

**DOI:** 10.1093/cdn/nzac094

**Published:** 2022-05-13

**Authors:** E Rochelle Werner, Charles D Arnold, Bess L Caswell, Lora L Iannotti, Chessa K Lutter, Kenneth M Maleta, Christine P Stewart

**Affiliations:** Institute for Global Nutrition, University of California, Davis, CA, USA; Institute for Global Nutrition, University of California, Davis, CA, USA; Institute for Global Nutrition, University of California, Davis, CA, USA; Western Human Nutrition Research Center, US Department of Agriculture, Davis, CA, USA; E3 Nutrition Lab, Washington University in St. Louis, St. Louis, MO, USA; RTI International, Washington, DC, USA; School of Global and Public Health, Kamuzu University of Health Sciences, Blantyre, Malawi; Institute for Global Nutrition, University of California, Davis, CA, USA

**Keywords:** eggs, iron, ferritin, soluble transferrin receptor (sTfR), hemoglobin, anemia, iron deficiency, inflammation, infant and child feeding, animal-source foods

## Abstract

**Background:**

Young children with diets lacking diversity with low consumption of animal source foods are at risk of iron deficiency anemia (IDA).

**Objectives:**

Our objectives were to determine the impact of supplementing diets with 1 egg/d on *1*) plasma ferritin, soluble transferrin receptor (sTfR), body iron index (BII), and hemoglobin concentrations and *2*) the prevalence of iron deficiency (ID), anemia, and IDA.

**Methods:**

Malawian 6–9-mo-old infants in the Mazira trial (clinicaltrials.gov; NCT03385252) were individually randomly assigned to receive 1 egg/d for 6 mo (*n* = 331) or continue their usual diet (*n* = 329). In this secondary analysis, hemoglobin, plasma ferritin, sTfR, C-reactive protein (CRP), and α-1-acid glycoprotein (AGP) were measured at enrollment and 6-mo follow-up. Iron biomarkers were corrected for inflammation. Ferritin, sTfR, BII, and hemoglobin were compared between groups using linear regression. Prevalence ratios (PRs) for anemia (hemoglobin <11 g/dL) and ID (ferritin <12 µg/L, sTfR >8.3 mg/L, or BII <0 mg/kg) between groups were compared using log binomial or modified Poisson regression.

**Results:**

A total of 585 children were included in this analysis (Egg: *n* = 286; Control: *n* = 299). At enrollment, the total prevalence of anemia was 61% and did not differ between groups. At 6-mo follow-up, groups did not differ in geometric mean concentration of hemoglobin [mean (95% CI); Egg: 10.9 (10.7, 11.1) g/dL; Control: 11.1 (10.9, 11.2) g/dL] and inflammation-adjusted ferritin [Egg: 6.52 (5.98, 7.10) µg/L; Control: 6.82 (6.27, 7.42) µg/L], sTfR [Egg: 11.34 (10.92, 11.78) mg/L; Control: 11.46 (11.04, 11.89) mg/L] or BII [Egg: 0.07 (0.06, 0.09) mg/kg; Control: 0.07 (0.05, 0.08) mg/kg]. There were also no group differences in anemia [Egg: 46%; Control 40%; PR: 1.15 (95% CI: 0.96, 1.38)], ID [PR: 0.99 (0.94, 1.05)], or IDA [PR: 1.12 (0.92, 1.36)].

**Conclusions:**

Providing eggs daily for 6 mo did not affect iron status or anemia prevalence in this context. Other interventions are needed to address the high prevalence of ID and anemia among young Malawian children. This trial is registered at http://www.clinicaltrials.gov as NCT03385252.

## Teaser

The egg intervention did not impact children's iron status or prevalence of anemia. Young children in rural Malawi remain at high risk of iron deficiency and anemia.

## Introduction

Iron deficiency (ID) is a major underlying cause for anemia, which can lead to impaired motor and cognitive development in children (1–3). Globally, half of children under 5 y of age have anemia, and one-quarter of the world's children are estimated to have iron deficiency anemia (IDA) (4). In a 2015–2016 survey of Malawian children under 2 y of age, 45% had anemia and 25% had IDA (5). Young children are at high risk for ID and anemia because they have high nutrient needs to support rapid growth (6) and the foods prepared for young children often lack adequate nutrient density. Diversifying diets of young children by including more animal-source foods can help children reach their nutrient requirements.

Eggs are a nutrient-dense food with potential to improve the dietary adequacy of many nutrients for young children (7, 8). However, the potential impact of eggs on iron status is unclear. One chicken egg contains 0.9 mg of non-heme iron (9), equivalent to 8% of the RDA for infants 6–12 mo old (11 mg/d) or 13% of the RDA for children 1–3 y of age (7 mg/d) (10). In eggs, iron is primarily concentrated in the yolk (11), with traces found in ovotransferrin in the egg whites (12). The iron content in eggs has limited bioavailability (13) because it is tightly bound to phosvitin (11), which is not readily degraded by proteolytic enzyme digestion (14). Moreover, whole eggs and egg whites inhibit iron bioavailability (15–17), reducing dietary absorption in adults by up to 27% (18). Nevertheless, 1 study from Australia has shown some potential for eggs to increase iron status among infants. After providing 4 egg yolks/wk to 6-mo-old infants for 6 mo, plasma iron and transferrin saturation were higher in the egg yolk group compared with the non-intervention control, but concentrations of ferritin and hemoglobin were similar between groups (19). Thus, the net effect of providing a whole egg to young children is unknown, and particularly to children living in areas with a high burden of ID and inflammation.

We recently conducted a study, entitled the Mazira Project, evaluating the impact of 1 egg/d on early child growth and development in Malawi (20). In this secondary analysis, we aimed to evaluate the impact of providing 1 egg/d to young children on indicators of iron status and anemia. We hypothesized that the egg intervention group would have higher mean concentrations of hemoglobin and ferritin, lower mean soluble transferrin receptor (sTfR), and lower prevalence of ID, anemia, and IDA as compared with the control group after the intervention period.

## Methods

### Study design, participants, and sample size

The Mazira Project was conducted between February 2018 and January 2019 in the Mangochi District of Malawi (clinicaltrials.gov registry NCT03385252). Children were randomly assigned to an intervention group, receiving 1 egg/d for 6 mo, or a control group that did not receive additional eggs. Details of the study design have been reported previously (20, 21). The study was promoted through community outreach events and study participants were recruited by home visits from household listings. Children were eligible if they were between the ages of 6.0 and 9.9 mo, were of singleton birth, and planned to reside in the catchment areas of the Lungwena or Malindi health centers for the study duration. Children were excluded based on wasting (midupper arm circumference ≤12.5 cm), severe anemia (hemoglobin ≤5 g/dL), bipedal edema, acute illness warranting hospital referral, history of egg allergy, congenital defects, or other morbidities that may impede growth or development. Children were referred to a health center if they presented with signs of severe dehydration or screened positive for wasting, bipedal edema, malaria, or severe anemia during any study visits.

Caregivers were oriented to the clinic facilities, activities, purpose, and procedures of the research study and had opportunities to ask questions and discuss concerns in a group setting and privately with staff members. They provided written informed consent at enrollment by signature or thumbprint to confirm their study participation, consent to future use of collected blood samples, and right to withdraw at any time. This study followed principles of ethical conduct approved by the Institutional Review Board at the University of California, Davis and the Research Ethics Committee at the University of Malawi College of Medicine.

### Randomization and masking

The target sample size for the main trial was 662 children, based on the desire to detect a 0.25 SD difference between groups in the primary outcome measure of length-for-age *z*-score with α = 0.05 and 80% power. Children were block-randomized in groups of 10 and allocated to the egg intervention or non-intervention group in a 1:1 ratio after enrolling and completing baseline assessments. From the current block, caregivers randomly selected 1 opaque, unmarked envelope containing a card with a unique randomization code to reveal their group assignment. Study staff conducting assessments were masked to group assignments.

### Intervention

A full description of the intervention groups has been published elsewhere (20). Briefly, each week caregivers in the egg intervention group received 7 eggs to feed the enrolled child 1 egg/d, plus 7 additional eggs to share with other household members. Study staff delivered eggs to intervention households twice per week and conducted recalls on the most recent egg feeding. The control group continued their usual diet, and their households were visited twice per week to report on the child's most recent meal. They received wash tubs, buckets, and plastic bins as participation incentives during the study and a mixed basket of foods, including eggs, at the completion of the study. This package of goods was selected to be of equal value to that of the eggs provided to the intervention group. All study participants received fabric cloth, sugar, and soap tablets after completing each visit.

### Data collection

During the initial clinic visit and 6-mo follow-up, children were assessed for growth, development, and dietary intake. Child recumbent length and weight were converted to *z*-scores using the sex- and age-specific WHO Growth Standards (22). Enrollment surveys and initial household visits assessed demographic characteristics of the study child and household members, including household assets and food insecurity using the Household Food Insecurity Access Scale (HFIAS) (23) and home environment using the Home Observation for Measurement of the Environment (HOME) (24) indicator. At clinic visits, trained nurses collected venous blood samples to measure hemoglobin concentration using a portable spectrophotometer (Hemocue Hb 201; HemoCue, Inc.) and presence of malaria antigens using a rapid diagnostic test kit (SD Bioline Malaria Ag P.f/Pan; Abbott Diagnostics) with >85% sensitivity and ≥90% specificity for *Plasmodium falciparum* (25, 26). Blood samples were collected in lithium heparin tubes, immediately placed in a cooler with ice, centrifuged at ambient temperature for 15 min at 1040 ×* g*, and placed in aliquots on site. Plasma samples were temporarily stored at –20°C and transported in coolers at the end of each day to a storage freezer maintained at –80°C. Aliquots were shipped on dry ice to laboratories completing plasma analyses.

Plasma ferritin, sTfR, C-reactive protein (CRP), α-1-acid glycoprotein (AGP), and retinol binding protein (RBP) were measured by combined sandwich techniques with ELISA methods by the VitMin Lab (27). All analytes were measured from a single well containing 50–75 µL plasma for all children who provided a minimum of 450 µL plasma sample from blood draws. A 10% subset of samples was reanalyzed for quality assurance. Replicates of pooled plasma samples were run with each tray, and the CV for each indicator was calculated as the following: ferritin (2.3%), sTfR (3.6%), CRP (5.8%), and AGP (8.1%).

### Statistical analysis

A detailed statistical analysis plan was developed and posted (https://osf.io/vfrg7) prior to analysis and analyst unblinding. All data cleaning, management, and analyses were performed using de-identified data in Stata (version 15; StataCorp LLC) (28). Indices above the upper limit of detection were replaced by the maximum observed values, and indices below the lower limit of detection were replaced by zeroes and converted to half the limit of detection as needed for analytical models performed on the log scale.

Ferritin and sTfR were corrected for subclinical inflammation on the log-transformed scale using the Biomarkers Reflecting Inflammation and Nutritional Determinants of Anemia (BRINDA) regression approach. This method adjusts for elevated CRP and AGP above the lowest decile set by an external reference group of preschool-aged children (29, 30). Body iron index (BII) was calculated according to Cook's formula (31) by applying constants to the ratio of sTfR:ferritin using inflammation-adjusted values, such that the quantitative estimates of iron stores are indicated by positive values and the magnitude of iron deficit is depicted with negative values (32). Dichotomous variables were created for anemia (hemoglobin <11 g/dL), ID (ferritin <12 µg/L, sTfR >8.3 mg/L, or BII <0 mg/kg), and IDA (both anemia and ID) (33–35).

Descriptive statistics were calculated for demographic characteristics, iron indices, and inflammation (CRP >5 mg/L or AGP >1 g/L) (36) at enrollment by group assignment. Linear regression models assessed groupwise differences in mean concentrations of hemoglobin and inflammation-corrected ferritin, sTfR, and BII. The prevalences of anemia, ID, and IDA were compared by group assignment using prevalence ratios estimated using logistic regression with a logarithmic link function and prevalence differences estimated using linear probability models with heteroscedasticity-consistent SEs (37). Modified Poisson models were used when log binomial models failed to converge (38). Our primary inferences were drawn from minimally adjusted models that controlled for baseline values of the outcome variable. For fully adjusted models, covariates were selected based on a bivariate association with the outcome variable (*P* < 0.1) from the following set of *a priori* identified variables: child age, child sex, maternal education, household asset index, number of children under 5 in the household, month of assessment, blood processing time, and inflammation-adjusted retinol binding protein (RBP). Malaria was examined for inclusion as a covariate based on a bivariate association (*P* < 0.1) with hemoglobin and anemia but not for ferritin, sTfR, BII, ID, or IDA, since these indicators included malaria in the correction for inflammation.

Linear regression models were used to impute missing baseline values, which affected 11% of hemoglobin and 20% of ferritin, sTfR, CRP, and AGP covariates included in analytical models. Demographic variables were evaluated for bivariate associations with each biomarker and were used for imputation of missing baseline measures when they retained significance (*P* < 0.1) in multivariable linear regression models. Additional sensitivity analyses were conducted excluding children missing baseline data and imputing with the mean value. Participant characteristics of children lost to follow-up or missing outcome measures were compared to children with complete measures. We used an inverse probability of censoring-weighted approach to reweight the analytic sample to match the enrolled sample and then compared these results with those from the principal models.

## Results

Children were randomly assigned to either the egg intervention group (*n* = 331) or the control group (*n* = 329). At enrollment, sociodemographic characteristics were balanced by treatment group (**Table 1**). On average, children were 7.4 mo old and lived with 5 other household members. Twenty percent of mothers had completed primary education, and 46% of mothers were literate. Most households (78%) reported moderate or severe food insecurity. Almost all children were breastfeeding, and 29% reported consuming a flesh food during the 24 h preceding enrollment.

**TABLE 1:** Enrollment characteristics of children in the Mazira Project, Malawi, 2018–2019, by intervention group^1^

	Egg		Control	
Characteristics	*n*	Value	*n*	Value
Maternal				
Age, y	329	25.9 ± 6.7	325	26.1 ± 6.8
Education,^2^*n* (%)	331	78 (24)	329	54 (16)
Literacy, *n* (%)	322	161 (50)	321	134 (42)
Household				
Number of children under 5 y	319	1.7 ± 0.8	319	1.7 ± 0.8
Number of household members	321	5.8 ± 2.6	320	6.0 ± 2.7
Moderate or severe food insecurity,^3^*n* (%)	331	247 (75)	329	267 (81)
Child				
Age, mo	331	7.4 ± 1.2	329	7.3 ± 1.2
Female, *n* (%)	331	160 (48)	329	159 (48)
Breastfeeding, *n* (%)	330	329 (100)	329	329 (100)
Meat consumption reported in 24-h recall, *n* (%)	330	111 (34)	329	77 (23)
Prevalence of stunting (LAZ <-2), *n* (%)	331	44 (13)	329	46 (14)
Prevalence of underweight (WAZ <-2), *n* (%)	331	24 (7)	329	28 (9)
Prevalence of wasting (WLZ <-2), *n* (%)	331	3 (1)	329	4 (1)
Inflammation, *n* (%)				
CRP >5 mg/L	265	91 (34)	260	93 (36)
AGP >1 g/L	265	158 (60)	260	159 (61)
Positive malaria test (RDT), *n* (%)	299	38 (13)	296	37 (13)
Hemoglobin, g/dL	292	10.5 (9.5, 11.5)	290	10.6 (9.3, 11.5)
Plasma ferritin,^4^ µg/L	265	13.1 (7.5, 23.8)	260	15.1 (8.7, 26.7)
Plasma sTfR,^4^ mg/L	265	10.2 (8.0, 13.6)	260	9.5 (7.7, 12.3)
Body iron index,^4^ mg/kg	265	−0.6 (−3.0, 1.9)	260	0.2 (−2.3, 2.4)
Anemia (hemoglobin < 11g/dL), *n* (%)	292	175 (60)	290	178 (61)
Iron deficiency (ferritin <12 µg/L),^4^*n* (%)	265	121 (46)	260	102 (39)
Iron deficiency (sTfR >8.3 mg/L),^4^*n* (%)	265	186 (70)	260	176 (68)
Iron deficiency (body iron index <0 mg/kg),^4^*n* (%)	265	151 (57)	260	120 (46)
Any iron deficiency,^4^*n* (%)	265	209 (79)	260	196 (75)
Iron deficiency anemia,^4^*n* (%)	265	138 (52)	259	132 (51)

1 Values are *n* (%), mean ± SDs, or median (25th, 75th percentile). AGP, α-1-acid glycoprotein; CRP, C-reactive protein; LAZ, length-for-age *z*-score; RDT, rapid diagnostic test; sTfR, soluble transferrin receptor; WAZ, weight-for-age *z*-score; WLZ, weight-for-length *z*-score.

2 Percent completed primary or greater.

3 Food insecurity assessed using the Household Food Insecurity Access Scale (23).

4 Inflammation corrected using the BRINDA (Biomarkers Reflecting Inflammation and Nutritional Determinants of Anemia) approach (29, 30).

**FIGURE 1:**
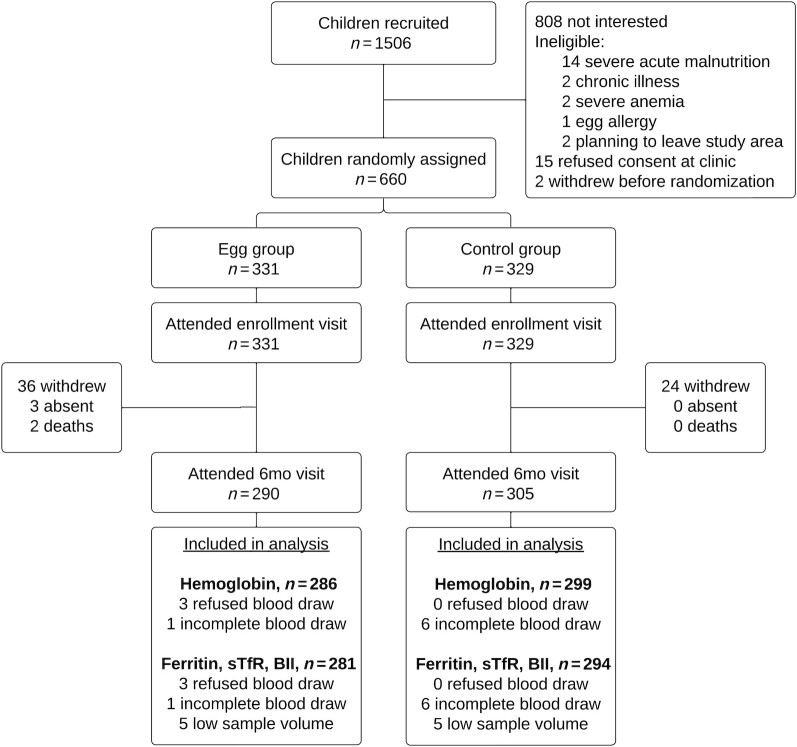
Participant flow diagram for the iron and anemia analyses of the Mazira Project, Malawi, 2018–2019. BII, body iron index; sTfR, soluble transferrin receptor.

After 6 mo of study participation, data were available on hemoglobin concentration from 585 children and iron status from 575 children (**Figure 1**). Overall, 13% of children had missing data due to study withdrawal, blood draw refusal, or insufficient sample volume, with similar rates of missing values in the egg intervention group (15%) and control group (11%). Compared with the baseline characteristics of participants with complete data, the participants excluded from analysis due to missing hemoglobin or iron biomarkers had mothers with lower levels of education and literacy and they lived in households with poorer quality housing, greater food insecurity, and rural residency (farming occupation and primary health care center). They were also enrolled earlier in the study (**Supplemental Table 1**).

At enrollment, 582 (88%) children completed a blood draw and were assessed for hemoglobin and 525 (80%) children provided a sufficient sample volume for assessment of inflammation and iron indices. Reasons for missing data did not differ by group: 4% refused consent, 8% incomplete blood draws, and 9% insufficient sample volume for analyses. Among participants assessed at enrollment, 61% were anemic, 35% had elevated CRP (>5 mg/L), 60% had elevated AGP (>1 mg/L), and 13% tested positive for malaria antigens. ID, as defined by 1 or more of the inflammation-adjusted iron biomarkers, affected 77% of children, and the overall prevalence of IDA was 52%. Iron indices and prevalence of ID at enrollment without inflammation-correction are listed in **Supplemental Table 2**.

After 6 mo of study participation, the prevalence of anemia declined to 43%, while the prevalence of ID increased to 89% (**Table 2**). Neither anemia nor ID differed by intervention group [anemia prevalence ratio (PR) (95% CI): 1.15 (0.96, 1.38); ID: 0.99 (0.94, 1.05)], and 93% of children with anemia were also iron deficient. The overall prevalence of positive tests for malaria antigens (6%), elevated CRP (28%), and elevated AGP (46%) did not differ between groups. There were also no groupwise differences in mean hemoglobin [geometric mean ratio (GMR) (95% CI): 0.99 (0.97, 1.01)], inflammation-adjusted ferritin [GMR (95% CI): 0.96 (0.85, 1.08)], sTfR [GMR (95% CI): 0.99 (0.94, 1.04)], and BII [GMR (95% CI): 1.06 (0.79, 1.42)]. Adjusting for additional covariates did not impact the groupwise comparisons. Findings did not differ in sensitivity analyses using inverse probability-weighted analysis or excluding children missing enrollment measures of hemoglobin or iron indices (data not shown).

**TABLE 2:** Difference in means and prevalence ratios of iron and anemia indices between treatment groups after 6 mo of participation in the Mazira Project, Malawi, 2018–2019^1^

	Egg		Control		GMR (95% CI)	
Variable	*n*	Geometric mean (95% CI)	*n*	Geometric mean (95% CI)	Minimally adjusted models^2^	Fully adjusted models^3^
Hemoglobin, g/dL	286	10.90 (10.74, 11.05)	299	11.06 (10.90, 11.21)	0.99 (0.97, 1.01)	0.98 (0.97, 1.00)
Plasma ferritin,^4^ µg/L	281	6.52 (5.98, 7.10)	294	6.82 (6.27, 7.42)	0.96 (0.85, 1.08)	0.96 (0.85, 1.07)
Plasma sTfR,^4^ mg/L	281	11.34 (10.92, 11.78)	294	11.46 (11.04, 11.89)	0.99 (0.94, 1.04)	0.99 (0.94, 1.05)
Body iron index,^4^ mg/kg	281	0.07 (0.06, 0.09)	294	0.07 (0.05, 0.08)	1.06 (0.79, 1.42)	1.05 (0.79, 1.40)
	*n*	%	*n*	%	PR (95% CI)	PR (95% CI)
Anemia (hemoglobin <11 g/dL), %	286	46	299	40	1.15 (0.96, 1.38)	1.18 (0.99, 1.42)
Any iron deficiency,^4^ %	281	90	294	89	0.99 (0.94, 1.05)	1.00 (0.94, 1.05)
Ferritin <12 µg/L,^4^ %	281	79	294	77	1.02 (0.93, 1.11)	1.02 (0.94, 1.11)
sTfR >8.3 mg/L,^4^ %	281	79	294	79	1.00 (0.92, 1.08)	1.00 (0.93, 1.08)
Body iron index <0 mg/kg,^4^ %	281	84	294	81	1.04 (0.96, 1.11)	1.04 (0.97, 1.12)
Iron deficiency anemia,^4^ %	281	42	294	37	1.12 (0.92, 1.36)	1.16 (0.95, 1.41)

1 GMR, geometric mean ratio; PR, prevalence ratio; sTfR, soluble transferrin receptor.

2 Adjusted for continuous baseline measures.

3 Adjusted for continuous baseline measures and covariates selected based on a bivariate association (*P* < 0.1) with the outcome among the following list: child sex, maternal education, number of children under 5 y in the household, month of assessment, minutes between blood collection and completion of aliquoting, and malaria (for hemoglobin and anemia only).

4 Inflammation corrected using the BRINDA (Biomarkers Reflecting Inflammation and Nutritional Determinants of Anemia) approach (29, 30).

## Discussion

In this study population of young children with a high prevalence of ID and anemia at enrollment that exceeded the WHO threshold (≥40%) for a problem of severe public health significance (33, 34), it is important to understand how a dietary intervention such as eggs may influence iron status. Eggs may inhibit bioavailability of iron in the diet, thus exacerbating the problem, or the provision of a small amount of iron through eggs could improve iron status. We found that providing eggs for daily consumption for 6 mo did not affect hemoglobin, ferritin, sTfR, or BII nor was there an effect on the prevalence of ID or anemia.

Our estimates of the prevalence of anemia and ID in the Mangochi District are similar to those reported in a national sample of young children included in the Malawi Micronutrient Survey in 2015–2016 (5). The prevalence of anemia among 6–23-mo-old children (45%) in the survey was similar to the prevalence of anemia among Mazira Project participants at study completion (43%). However, the Mazira Project participants were notably more iron deficient (76%) than the national average of 6–23-mo-old children (43%) following the same BRINDA linear regression approach and cutoff of inflammation-adjusted ferritin at <12 µg/L.

Iron requirements among infants and young children are high to meet the demand for rapid growth. Had this study found more rapid growth in the intervention group, there could have been even greater demand for iron than among control children. Nevertheless, the high burden of ID in both groups is likely due to multiple compounding factors, including low dietary intake of iron-rich foods, high intake of foods containing phytates that could inhibit iron absorption, and prevalence of inflammation and malaria. The usual mean intake of iron was 1.9 mg at enrollment, and after 6 mo of study participation, the usual mean intake of iron did not differ between the egg intervention group (3.0 mg) and control group (2.8 mg) (39). Inadequate iron intake was nearly ubiquitous among children at both time points. Maize is the staple food and predominant dietary source of phytates among Malawian infants. Legumes, leafy green vegetables, and tea were also commonly consumed (40); and the phytates, oxalates, and tannins in these foods may also impede iron absorption (32). Absorption of dietary iron is also reduced in response to sustained inflammatory response, which was highly prevalent among Mazira Project participants. At enrollment, most children had at least 1 elevated marker of inflammation, and 13% tested positive for malarial antigens.

The lack of effect of the egg intervention on iron indices among Malawian children somewhat contrasts with the results from an egg yolk intervention trial among Australian infants (19). That study provided 4 egg yolks/wk to 6-mo-old infants for 6 mo and found that the intervention significantly increased plasma iron (egg: 10.5 µmol/L; control: 8.3 µmol/L; *P* < 0.05) and transferrin saturation percentage points (egg: 14.3%; control 10.8%; *P* < 0.05) but had no impact on ferritin, transferrin, or hemoglobin. In comparison to Australian infants, Mazira Project participants assigned to the nonintervention control group had lower hemoglobin (11.1 vs. 12.0 g/dL), lower ferritin (6.8 vs. 20.6 µg/L), and greater prevalence of ID [77% (inflammation-adjusted ferritin <12 µg/L) vs. 17% (ferritin <10 µg/L)] after 6 mo of study participation. The differences in burden of ID and anemia may be explained by differential intake of dietary iron and prevalence of inflammation. At study completion, the breastfed Australian children in the control group consumed 6.9 ounces of flesh foods per week (or 28 g/d), whereas Malawian children in the control group consumed fewer flesh foods (estimated usual intake of 24 kcal/d, or 6 g/d assuming 4 kcal/g) and likely had lower total iron intake as well (39, 40). Inflammation was highly prevalent among Malawian study participants and was not reported in the Australian study. Despite differences in study context, neither study detected significant groupwise differences in hemoglobin or ferritin concentration. The Malawian study had a larger enrollment and was powered to detect smaller differences in mean hemoglobin and ferritin between groups than the Australian study. The Australian trial reported significant differences in plasma iron and transferrin saturation; however, the Mazira Project did not measure transferrin saturation or find groupwise differences in plasma iron (Lora Iannotti, Washington University in St. Louis, personal communication, 2021).

Prior short-term, single-meal studies in adults have shown the potential for eggs to inhibit absorption of nonheme iron from other foods in a meal (13, 15), which is hypothesized to be through the binding action of phosvitin and ovotransferrin (8, 14). However, the long-term effects of habitual consumption of eggs on iron bioavailability from the total diet have not been examined. Some studies of high-phytate diets on iron bioavailability have found that single-meal studies overestimate the inhibitory effect of phytates on iron absorption compared with longer-term, whole-diet assessments (41, 42). While our study was not designed to directly assess iron bioavailability, it is important to note that the egg intervention did not negatively impact infants’ iron status or exacerbate the ongoing problem of ID in this study sample.

This study had some limitations inherent to the selected measures included in analysis. Adherence would have been best evaluated through daily feeding observations; however, caregivers frequently fed eggs to their children in the early morning and it was only feasible to observe a portion of these feedings each week. Nevertheless, adherence was measured by caregiver report during the twice-weekly home visits as well as through 24-h dietary recalls at 3- and 6-mo follow-up. The reported consumption of eggs during 24-h recall interviews was higher in the egg group (3 mo: 85%; 6 mo: 71%) than the control group (3 mo: 7%; 6 mo: 7%) (20, 40). The usual energy intake from eggs at 3-mo and 6-mo follow-up was approximately 30 kcal/d in the egg group and 1 kcal/d in the control group (40). We also compared plasma metabolites between groups and noted several markers related to egg consumption differed between the egg intervention and control group (43). These biomarkers are less susceptible to reporting bias and suggest that the eggs were indeed consumed by the index child, but they cannot provide information on dose or frequency of egg consumption.

Eggs were fed to children according to the caregivers’ preference, reflecting real-world usual feeding practices instead of standardized cooking methods. This introduced greater variability in interactions between eggs and other nutrients or bioactive components of the food matrix, which could potentially affect iron absorption. A comprehensive suite of iron biomarkers would be needed to better understand iron absorption and metabolism in this study population with a high prevalence of dietary iron inadequacy, inflammation, and malaria. Data on enteric diseases, hemoglobinopathies, and other genetic conditions were not collected to determine multifaceted underlying etiologies of anemia. Nevertheless, since 93% of study participants with anemia had concurrent ID, it is apparent that ID is an important contributor to anemia in this context.

The high level of missing biomarker data from children at enrollment (12% hemoglobin; 20% iron indices) and 6-mo follow-up (11% hemoglobin; 13% iron indices) also presents a limitation and could have introduced selection bias. Conducting blood draws on young children presents several challenges, including difficulty in locating small superficial veins as well as caregivers’ consent to complete the blood draw despite the child's discomfort. A greater number of caregiver refusals were reported at enrollment than at 6-mo follow-up. Because we included baseline values as a covariate in our models, we imputed missing baseline values using multivariate linear regression. Results did not differ when compared with multiple sensitivity analyses.

The strengths of our study include its design as a randomized controlled trial, execution with high follow-up rates, and ability to detect small effect sizes with its large sample size. The Mazira Project had high adherence (>70% reported consuming eggs on the previous day) at the 3- and 6-mo follow-up (20). Analysis of dietary recalls revealed a high prevalence of inadequate iron intake and provides general agreement with the high prevalence of ID reported in this paper. The analysis of ferritin and sTfR met high standards for quality control, and the data analysis was conducted according to a prespecified statistical analysis plan. Therefore, it is likely that the lack of difference in measured iron and anemia status between the egg intervention and control groups accurately depicts the true effect.

We conclude that providing eggs did not affect ID or anemia prevalence among young children in a population with a high burden of these conditions. While eggs are rich in other nutrients, including choline, vitamin A, and essential amino acids, the promotion of eggs will not address the problem of ID among young children. One egg per day does not provide enough iron to meet the requirements in this population. Low iron stores from birth and iron-deficient diets put young children at increased risk for ID and anemia. The high burden of ID and anemia among Malawian infants and young children is concerning, and other interventions such as multiple micronutrient powders (44), lipid-based nutrient supplements (45), or promotion of other iron-rich foods such as small fish or chicken liver (46–48) are needed to address these issues. Additionally, any future nutrition-related interventions designed to address ID and anemia in this population are recommended to be implemented in conjunction with measures to control malaria and reduce inflammation.

## Supplementary Material

nzac094_Supplemental_File

## Data Availability

Data described in the manuscript, code book, and analytic code will be made publicly and freely available without restriction at https://osf.io/vfrg7.

## Notes

Supported by the Bill and Melinda Gates Foundation (award number: OPP1169033).

Author disclosures: The authors report no conflicts of interest. The funder provided input on the design of the study but was not involved in the conduct, analysis, or reporting of results.

Supplemental Tables 1 and 2 are available from the “Supplementary data” link in the online posting of the article and from the same link in the online table of contents at https://academic.oup.com/cdn/.

Abbreviations used: AGP, α-1-acid glycoprotein; BII, body iron index; BRINDA, Biomarkers Reflecting Inflammation and Nutritional Determinants of Anemia; CRP, C-reactive protein; GMR, geometric mean ratio; ID, iron deficiency; IDA, iron deficiency anemia; PR, prevalence ratio; RBP, retinol binding protein; sTfR, soluble transfer receptor.
