# Supplementary material for: The Effects of 1 Egg per Day on Iron and Anemia Status among Young Malawian Children: A Secondary Analysis of a Randomized Controlled Trial
Source: Curr Dev Nutr. 2022 May 13;6(6):nzac094. doi: 10.1093/cdn/nzac094 (PMC9213210; doi:10.1093/cdn/nzac094)
Supplement: nzac094_Supplemental_File [file nzac094_supplemental_file.docx]

### The Effects of One Egg per Day on Iron and Anemia Status among Young Malawian Children: A Secondary Analysis of a Randomized Controlled Trial

*E. Rochelle Werner^1^, Charles D. Arnold^1^, Bess L. Caswell^1,2^, Lora L. Iannotti^3^, Chessa K. Lutter^4^, Kenneth M. Maleta^5^, Christine P. Stewart^1^*

^1^Institute for Global Nutrition, University of California, Davis, Davis, CA, USA

^2^Western Human Nutrition Research Center, U.S. Department of Agriculture, Davis, CA, USA

^3^E3 Nutrition Lab, Washington University in St. Louis, St. Louis, MO, USA

^4^RTI International, Washington, DC, USA

^5^School of Global and Public Health, Kamuzu University of Health Sciences, Blantyre, Malawi

Online Supplementary Material

### Supplementary Table 1: Characteristics of participants missing iron indices at 6mo follow-up of the Mazira Project, Malawi, 2018-2019

|  | Missing (n=85) | |  | Complete (n=575) | |  |
| --- | --- | --- | --- | --- | --- | --- |
| Characteristic | n^1^ | % or mean ± SD |  | n^1^ | % or mean ± SD | p-value |
| Maternal age, y | 79 | 25.2 ± 7.4 |  | 575 | 26.1 ± 6.6 | 0.276 |
| Maternal education (% completed primary or greater) | 85 | 12 |  | 575 | 21 | **0.046** |
| Maternal literacy (% can read) | 71 | 37 |  | 572 | 47 | **0.099** |
| Maternal tribe |  |  |  |  |  |  |
| Chewa or other | 71 | 10 |  | 572 | 15 | 0.246 |
| Yao |  | 90 |  |  | 85 |  |
| Maternal occupation |  |  |  |  |  |  |
| Farming | 71 | 59 |  | 571 | 41 | **0.0128** |
| Service |  | 13 |  |  | 25 |  |
| Housewife |  | 28 |  |  | 34 |  |
| Paternal occupation |  |  |  |  |  |  |
| Farming or Fishing | 58 | 41 |  | 458 | 49 | 0.254 |
| Service |  | 59 |  |  | 51 |  |
| Muslim religion | 71 | 92 |  | 572 | 88 | 0.335 |
| Health center |  |  |  |  |  |  |
| Lungwena | 85 | 71 |  | 575 | 51 | **0.001** |
| Malindi |  | 29 |  |  | 49 |  |
| Poor floor quality^2^ | 71 | 83 |  | 572 | 76 | 0.168 |
| Poor roof quality^2^ | 71 | 72 |  | 572 | 60 | **0.049** |
| Poor wall quality^2^ | 71 | 54 |  | 572 | 43 | **0.084** |
| Household assets |  |  |  |  |  |  |
| HOME inventory score^3^ | 71 | 24 ± 3.1 |  | 572 | 24.2 ± 3.6 | 0.669 |
| own latrine | 71 | 96 |  | 572 | 97 | 0.755 |
| own cows | 71 | 4 |  | 572 | 3 | 0.506 |
| own goats | 83 | 13 |  | 575 | 20 | 0.157 |
| own chickens | 84 | 35 |  | 575 | 32 | 0.644 |
| Number of children under 5 y | 70 | 1.7 ± 0.8 |  | 568 | 1.7 ± 0.8 | 0.845 |
| Number of household members | 70 | 5.8 ± 3.1 |  | 571 | 5.9 ± 2.6 | 0.718 |
| Moderate or severe food insecurity^4^ | 85 | 86 |  | 575 | 77 | **0.060** |
| Child |  |  |  |  |  |  |
| child age, mo | 85 | 7.5 ± 1.2 |  | 575 | 7.4 ± 1.2 | 0.199 |
| female, % | 85 | 47 |  | 575 | 49 | 0.801 |
| prevalence of stunting (LAZ<-2) | 85 | 13 |  | 575 | 14 | 0.841 |
| prevalence of underweight (WAZ<-2) | 85 | 6 |  | 575 | 8 | 0.466 |
| prevalence of wasting (WLZ<-2) | 85 | 1 |  | 575 | 1 | 0.911 |
| prevalence of malaria | 71 | 11 |  | 524 | 13 | 0.718 |
| LAZ = length-for-age z-score; WAZ = weight-for-age z-score; WLZ = weight-for-length z-score ^1^Number of children with data at enrollment or first household visit  ^2^Poor quality defined as straw, grass, mud, or unburnt brick  ^3^HOME, Home Observation for Measurement of the Environment (24)  ^4^Food insecurity assessed using Household Food Insecurity Access Scale (23) | | | | | | |

### Supplementary Table 2: Iron indices for children in the Mazira Project, Malawi, 2018-2019, by intervention group at enrollment without correction for inflammation

|  | Egg (n=265) | Control (n=260) |
| --- | --- | --- |
| Characteristic | n (%) or median (P25, P75) | n (%) or median (P25, P75) |
| Plasma ferritin, µg/L | 23.8 (12.4, 49.86) | 28.0 (15.5, 53.09) |
| Plasma sTfR, mg/L | 11.6 (9.1, 16.2) | 11.0 (8.8, 14.6) |
| Body iron index, mg/kg | 1.1 (-1.6, 3.6) | 1.9 (-0.7, 4.3) |
| Iron deficiency (ferritin < 12µg/L), % | 64 (24) | 44 (17) |
| Iron deficiency (sTfR > 8.3mg/L), % | 227 (86) | 210 (81) |
| Iron deficiency (body iron index < 0mg/kg), % | 107 (40) | 74 (28) |
| Any iron deficiency, % | 234 (88) | 212 (82) |
| Iron deficiency anemia, % | 148 (56) | 139 (54) |
| sTfR = soluble transferrin receptor | | |
